# Supplementary material for: Genetic determinants of fatigue up to 2 years after radiotherapy in prostate cancer patients
Source: Nat Commun. 2026 Apr 22;17:3703. doi: 10.1038/s41467-026-72041-3 (PMC13102951; doi:10.1038/s41467-026-72041-3)
Supplement: Supplementary file 1 — Supplementary information [file 41467_2026_72041_MOESM1_ESM.pdf]

Supplementary information

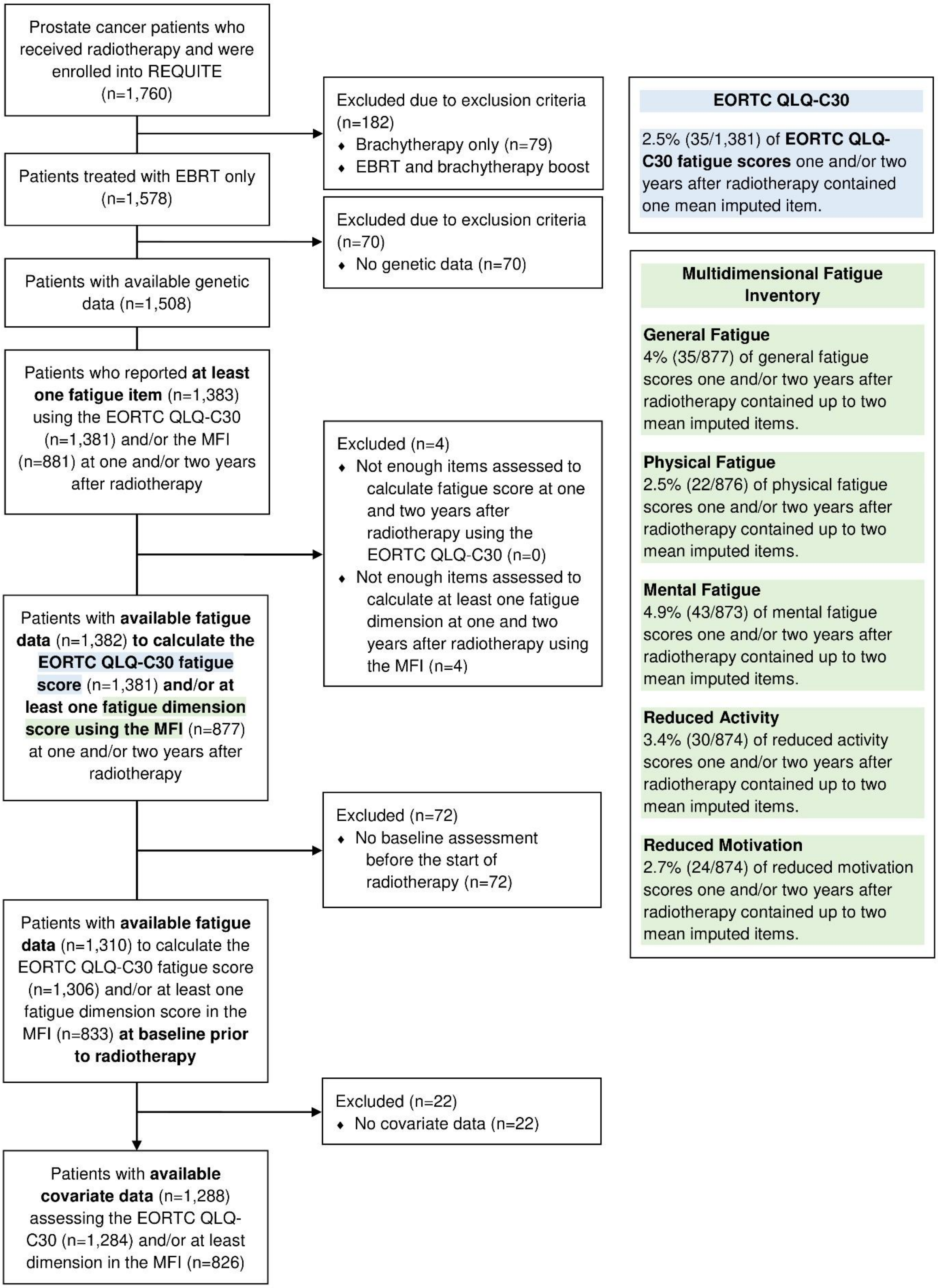

Supplementary figure 1. Flow diagram on the availability of long-term fatigue assessments using the EORTC QLQ-C30 and Multidimensional Fatigue Inventory up to two years after radiotherapy in prostate cancer patients of the REQUITE cohort.

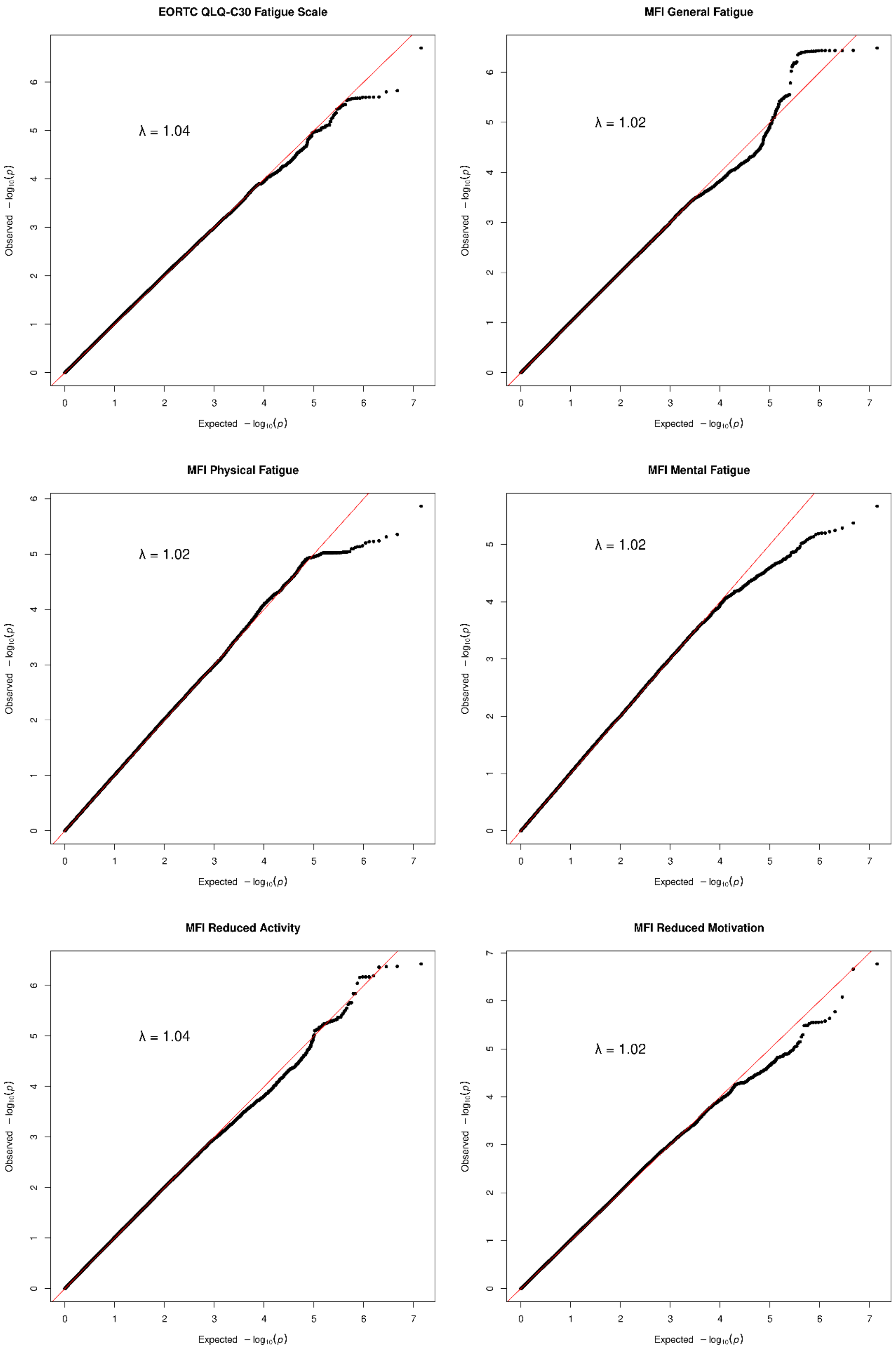

Supplementary figure 2. QQ plots for fatigue in the EORTC QLQ-C30 (N=1,284) and distinct fatigue dimensions in the Multidimensional Fatigue Inventory (N=826) up to two years following external beam radiotherapy in prostate cancer patients of the REQUITE cohort.

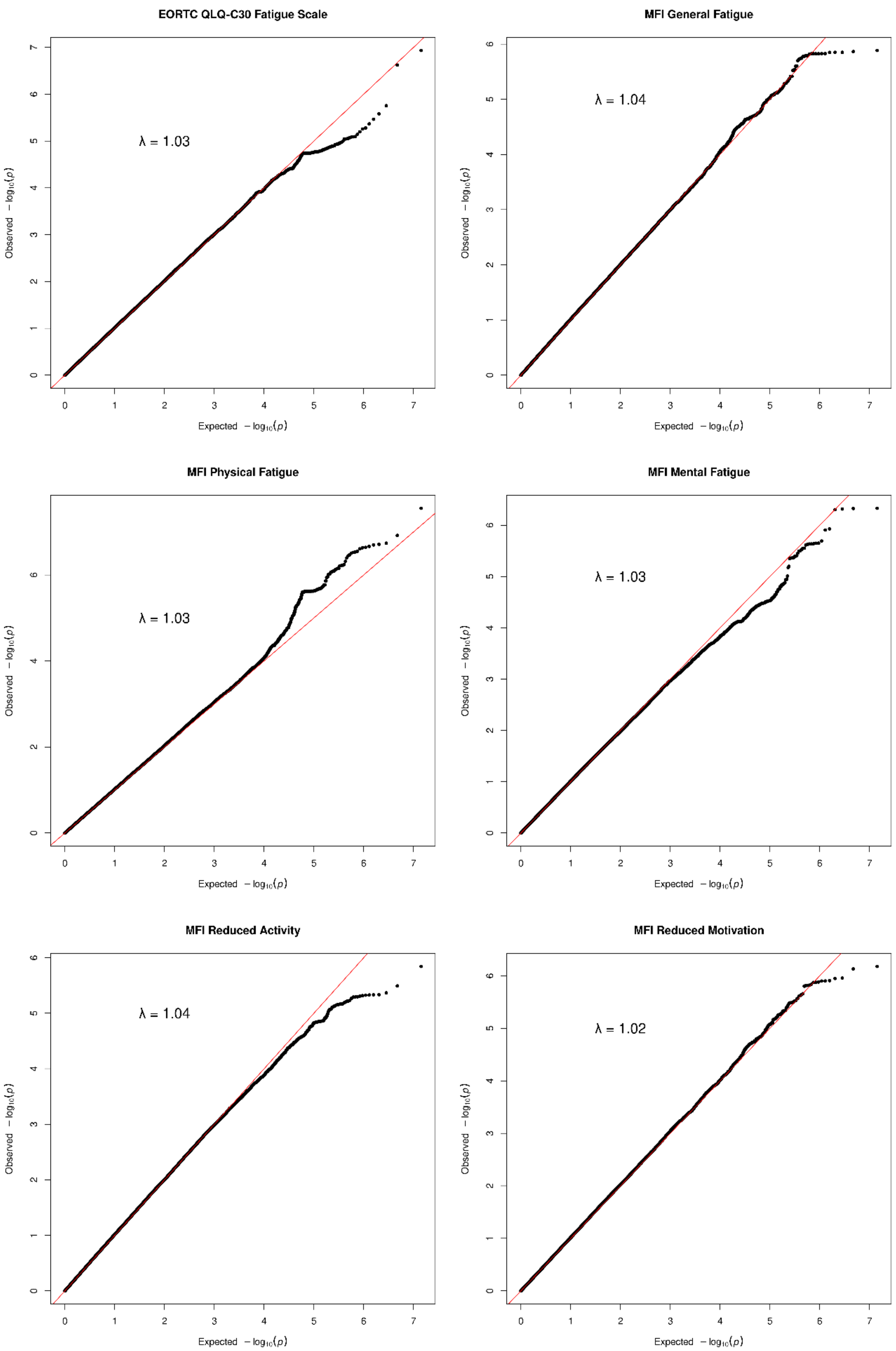

Supplementary figure 3. QQ plots for fatigue in the EORTC QLQ-C30 (N=1,147) and distinct fatigue dimensions in the Multidimensional Fatigue Inventory (up to N=677) up to two years following external beam radiotherapy in prostate cancer patients of the REQUITE cohort without fatigue before the start of radiotherapy.

Supplementary table 1. Odds ratios and 95% confidence intervals for the index SNP rs142212041 in the genome-wide association subgroup and sensitivity analyses on clinically important incident physical fatigue in the MFI up to two years after the end of radiotherapy in prostate cancer patients of the REQUITE cohort without clinically important fatigue before radiotherapy.

	Odds Ratio (95% Confidence Interval)
Subgroup Analyses [1]	
**Receipt of ADT**	
No ADT (N=207)	2.53 (1.32-4.84)
Any ADT (N=436)	3.52 (2.15-5.76)
**Receipt of Pelvic Radiotherapy**	
No Pelvic Radiotherapy (N=397)	3.20 (2.01-5.10)
Pelvic Radiotherapy (N=246)	3.09 (1.44-6.65)
**Time Point of Fatigue Assessment**	
1 Year Post-Radiotherapy (N=603)	2.05 (1.30-3.23)
2 Years Post-Radiotherapy (N=548)	3.29 (2.08-5.20)
**More Restrictive Threshold of Clinical Importance**[2] (N=755)	2.26 (1.42-3.61)
**Complete MFI Physical Fatigue Assessments** (N=620)	3.05 (2.04-4.55)
Sensitivity Analyses [1]	
**Reduced Covariate Set**[3] (N=643)	2.92 (1.99-4.29)
**Extended by five additional genetic ancestry principal components** (N=643)	2.98 (2.02-4.42)

[1] N corresponds to the number of patients included in the regression model.
[2] The threshold for clinically important fatigue was raised from scores ≥75th to scores ≥90th percentile in the general German male population aged ≥60 years [24].
[3] The reduced covariate set was derived by fitting a logistic regression model for incident physical fatigue up to two years after radiotherapy, adjusting for the covariates (excluding genetic data) listed in the methods section. Covariates with *P*≤0.2 (age, BMI, tumour size) were included in the reduced covariate set.

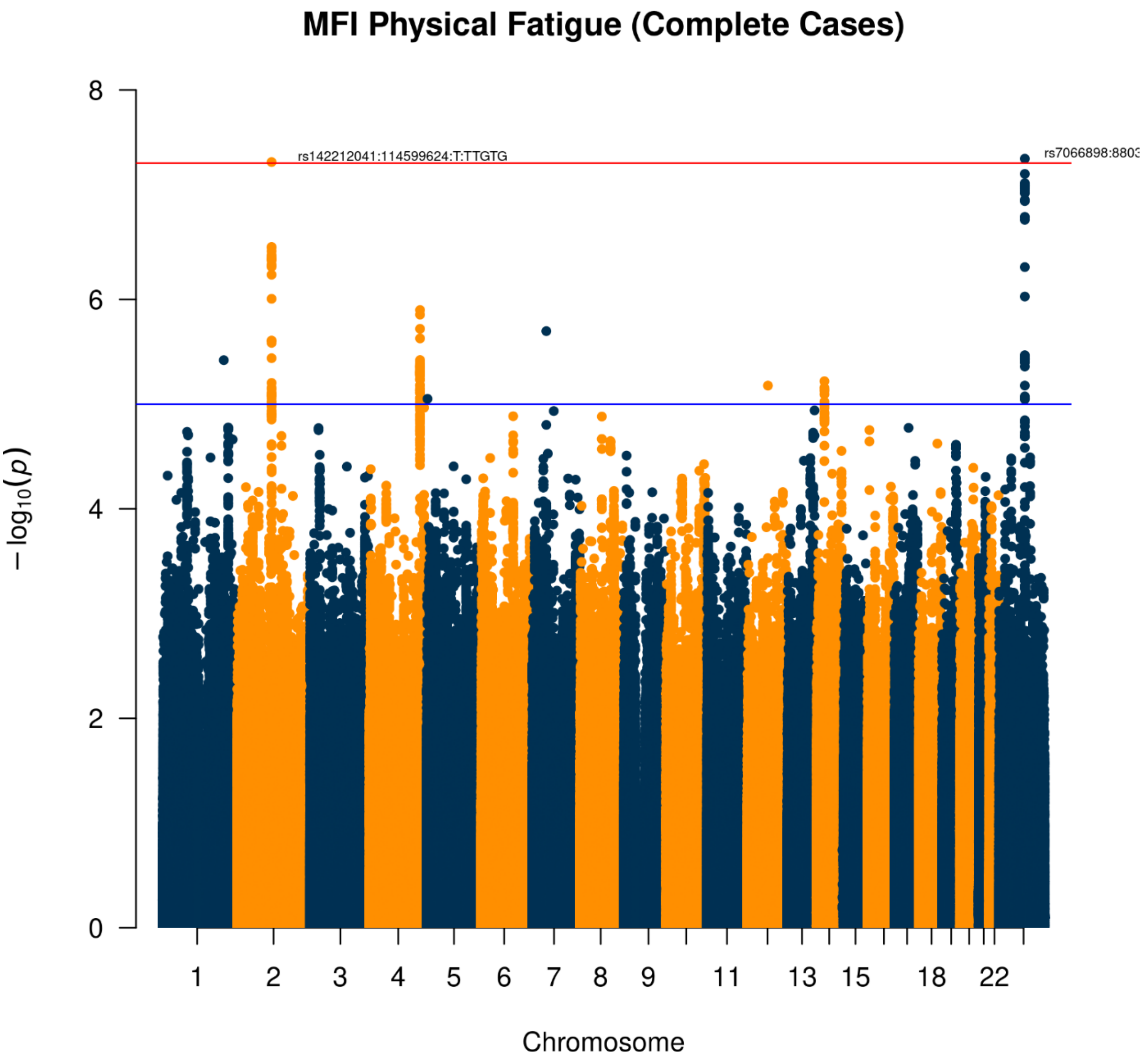

Supplementary figure 4. Manhattan plot for physical fatigue in the Multidimensional Fatigue Inventory (N=620) up to two years following external beam radiotherapy in prostate cancer patients of the REQUITE cohort without fatigue before the start of radiotherapy who provided complete MFI assessments for the physical fatigue dimension. Multivariable regression models were adjusted for age and BMI at enrolment, baseline fatigue level prior to radiotherapy, depression, hormone therapy, prostatectomy, tumour size, nodal involvement, pelvic radiotherapy, and the top 5 principal components. The red line indicates genome-wide statistical significance (P<5×10-8) and the blue line indicates suggestive significance ($P<5\times10^{-6}$).

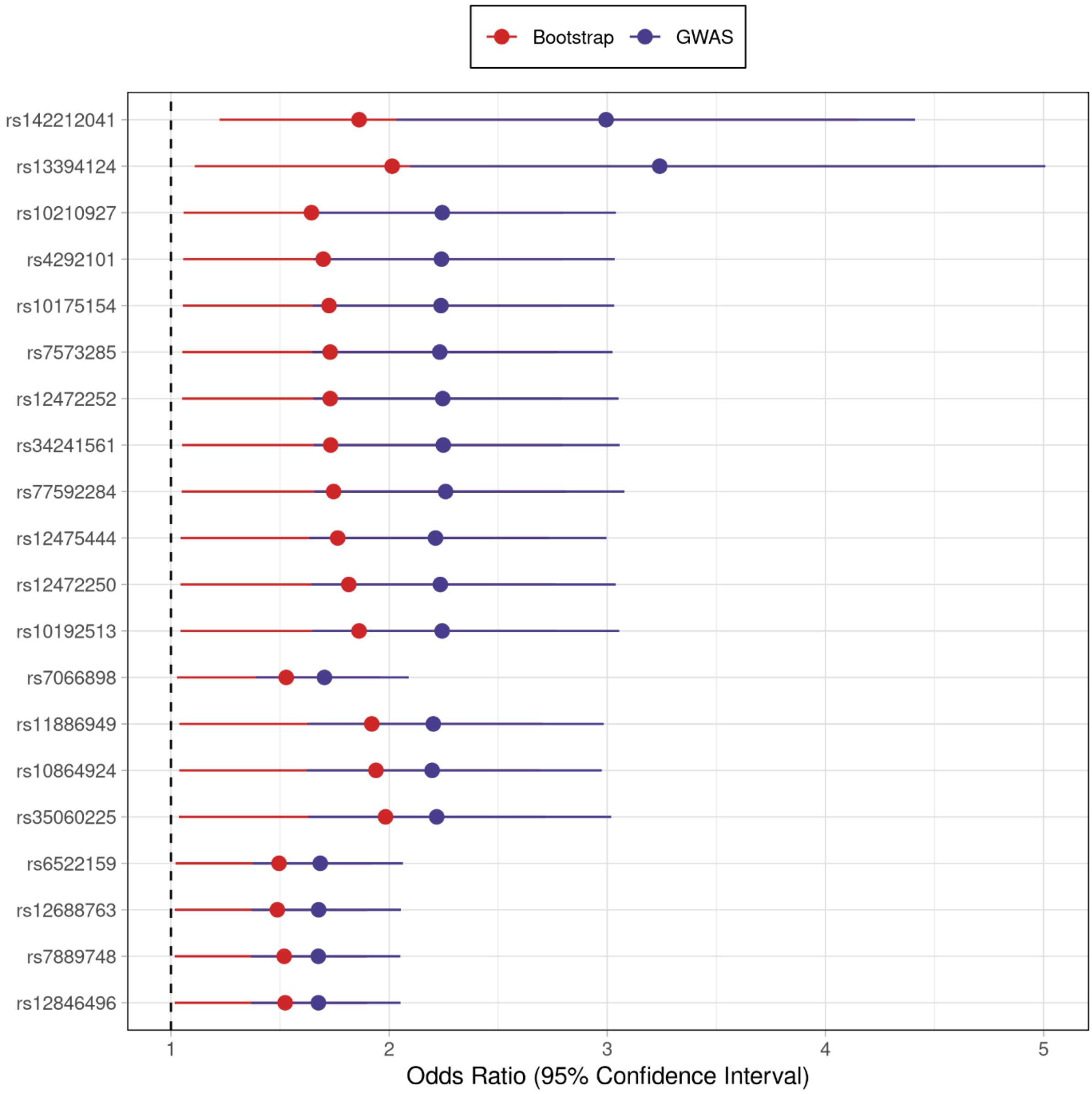

Supplementary figure 5. Bootstrap (3,000 replicates) and GWAS-derived odds ratios and 95% confidence intervals for the top 20 SNPs in the genome-wide association analyses on clinically important incident physical fatigue in the MFI up to two years after the end of radiotherapy in prostate cancer patients of the REQUITE cohort without clinically important fatigue before radiotherapy.

Supplementary table 2. Odds ratios and 95% confidence intervals for the SNP rs142212041 in the genome-wide association analyses on clinically important incident physical fatigue in the MFI up to two years after the end of radiotherapy in prostate cancer patients of the REQUITE cohort without clinically important fatigue before radiotherapy excluding one country respectively.

	Odds Ratio (95% Confidence Interval) rs142212041
**Leave-One-Out Analysis[1]**	
Excluding France (N=487)	2.81 (1.80-4.38)
Excluding Germany (N=593)	3.44 (2.29-5.16)
Excluding Italy (N=524)	3.04 (1.99-4.63)
Excluding Spain (N=464)	2.30 (1.39-3.81)
Excluding United Kingdom (N=509)	3.22 (2.10-4.92)
Excluding United States (N=638)	2.96 (2.01-4.37)

[1] N corresponds to the number of patients included in the regression model.

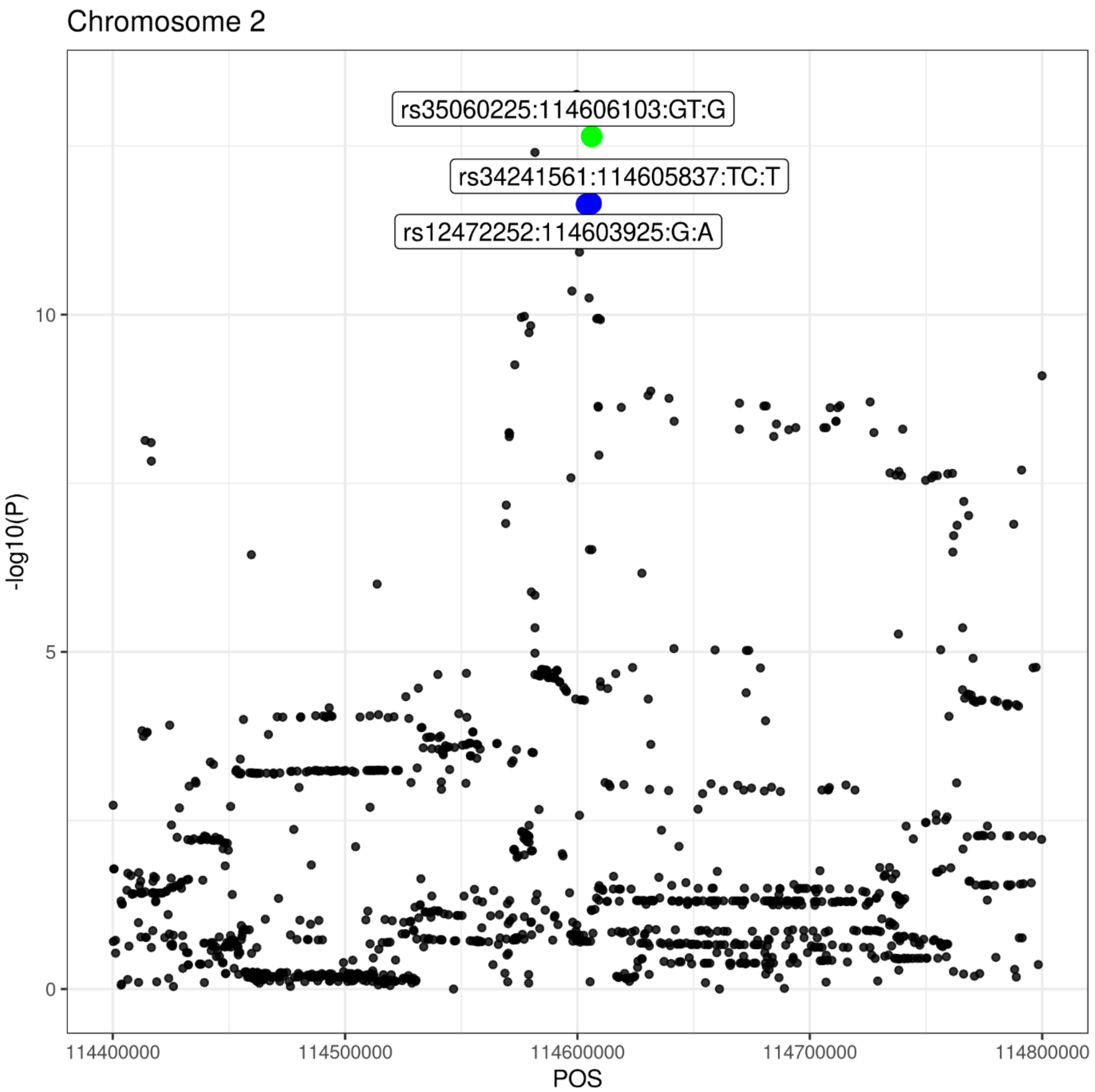

Supplementary figure 6. Locus plot for the interval spanning 114.40 Mb to 114.80 Mb on chromosome 2 highlighting the genomic position of the candidate causal variants.

eQTpLot analysis for Incident Physical Fatigue and ACTR3 In Muscle - Skeletal

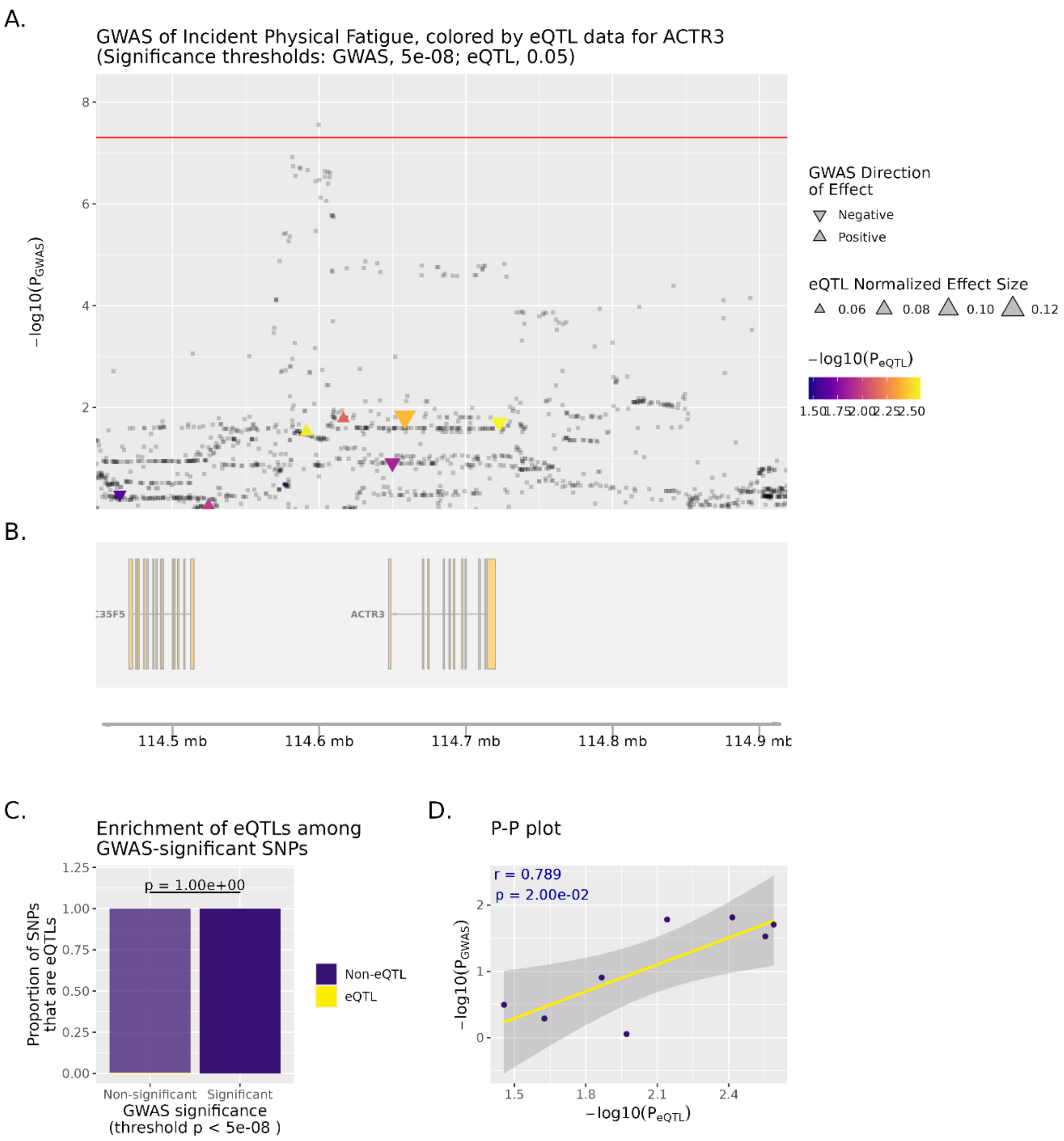

Supplementary figure 7. eQTL-GWAS colocalisation plot including eQTL enrichment and P-P correlation for the locus with the genome-wide statistically significant SNP rs142212041 and 200kb each around the candidate gene *ACTR3* based on the genome-wide association analysis for incident physical fatigue and skeletal muscle tissue eQTLs (GTEx v7).

eQTpLot analysis for Incident Physical Fatigue and ACTR3 In Whole Blood

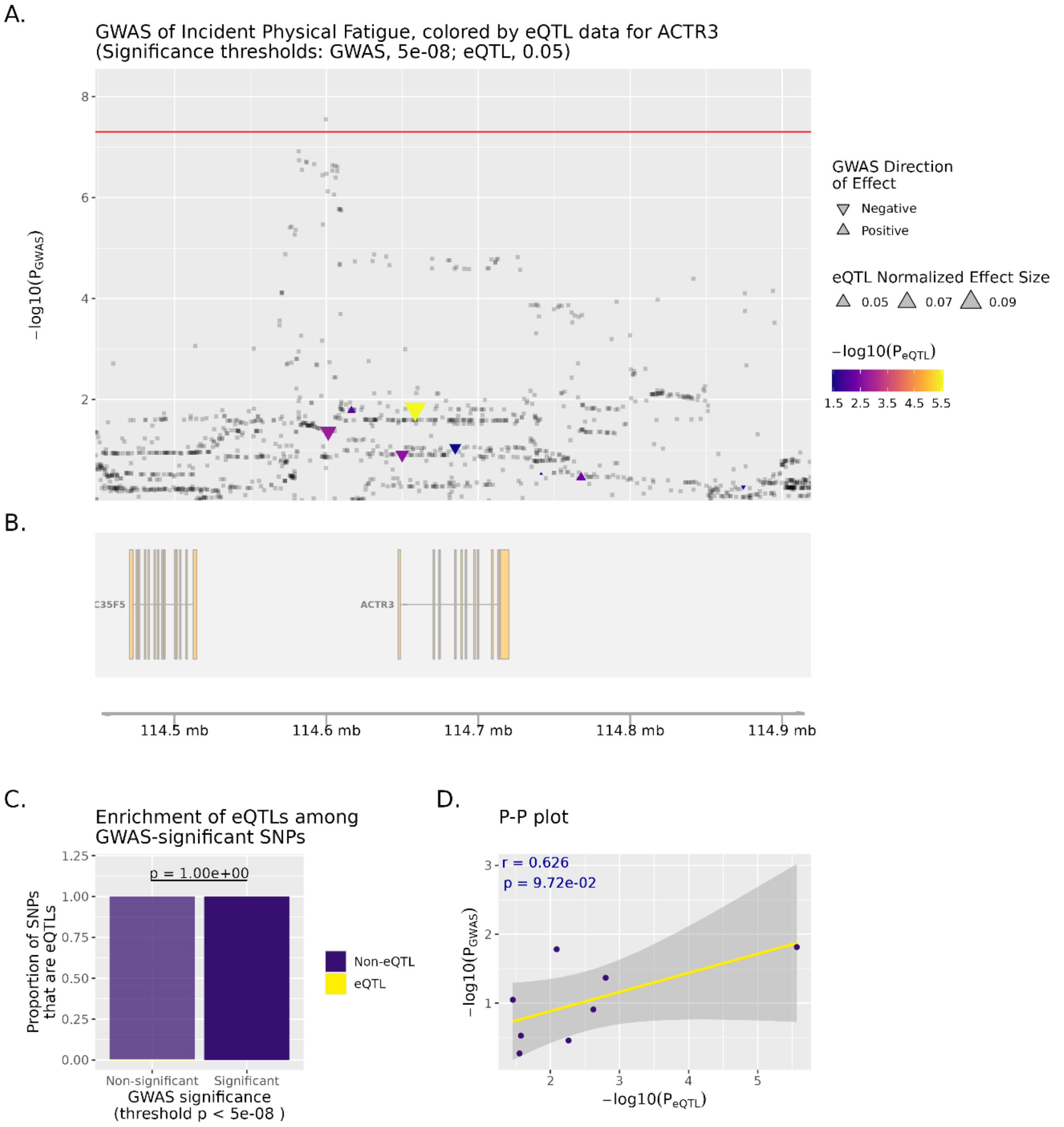

Supplementary figure 8. eQTL-GWAS colocalisation plot including eQTL enrichment and P-P correlation for the locus with the genome-wide statistically significant SNP rs142212041 and 200kb each around the candidate gene *ACTR3* based on the genome-wide association analysis for incident physical fatigue and whole blood eQTLs (GTEx v7).

eQTpLot analysis for Incident Physical Fatigue and ACTR3 In Pituitary

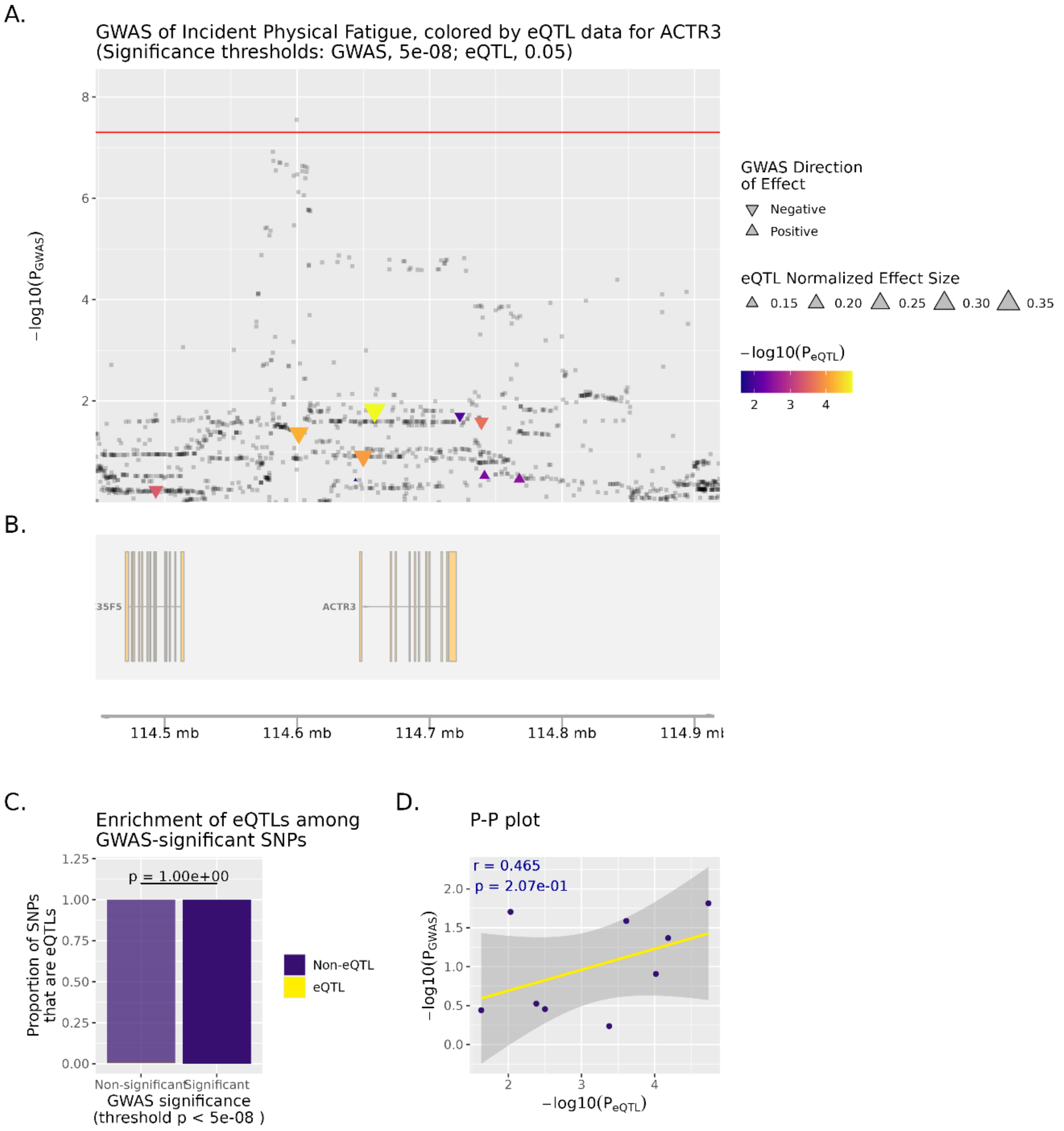

Supplementary figure 9. eQTL-GWAS colocalisation plot including eQTL enrichment and P-P correlation for the locus with the genome-wide statistically significant SNP rs142212041 and 200kb each around the candidate gene *ACTR3* based on the genome-wide association analysis for incident physical fatigue and pituitary gland eQTLs (GTEx v7).

eQTpLot analysis for Incident Physical Fatigue and ACTR3 In Brain - Hippocampus

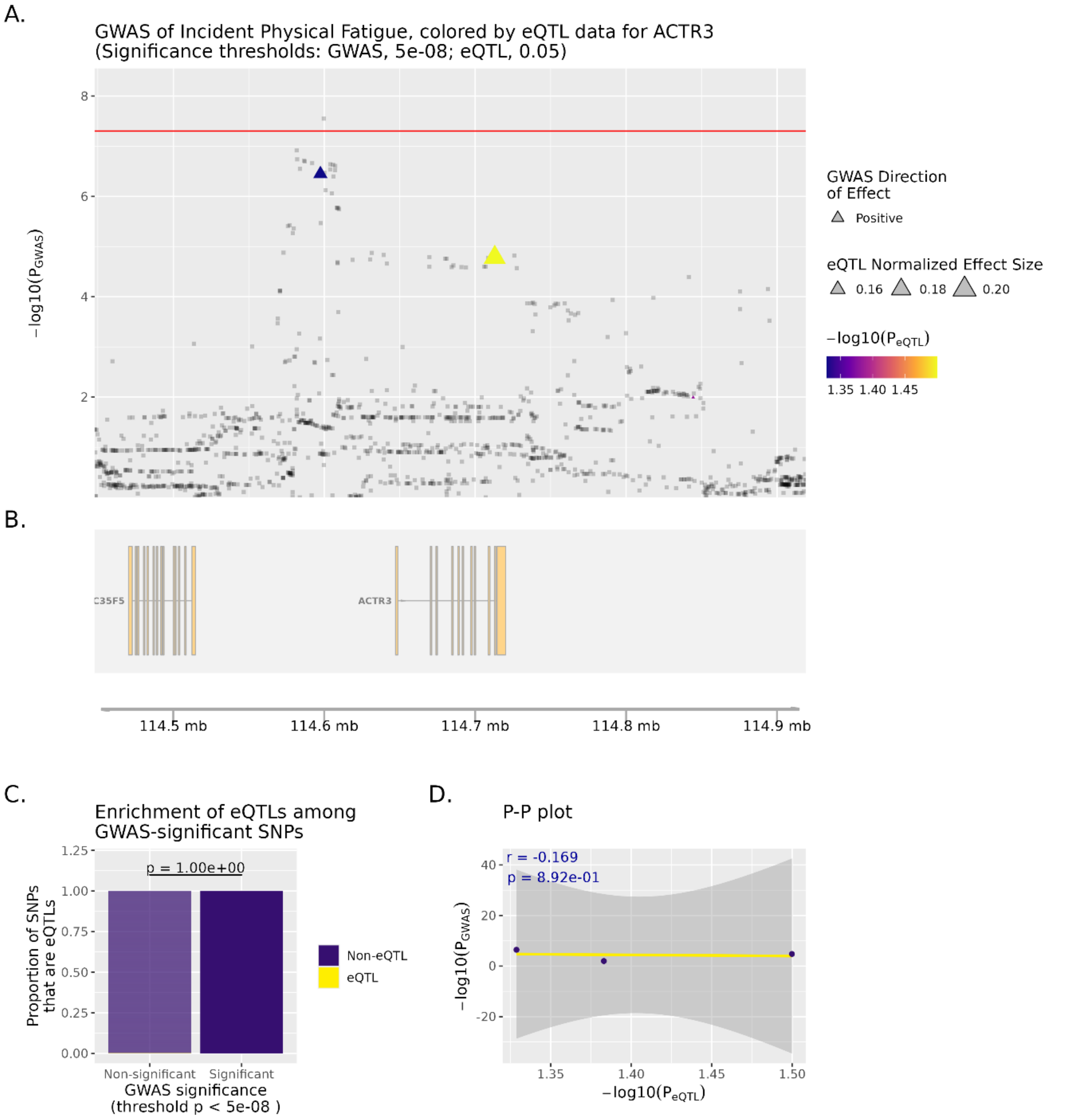

Supplementary figure 10. eQTL-GWAS colocalisation plot including eQTL enrichment and P-P correlation for the locus with the genome-wide statistically significant SNP rs142212041 and 200kb each around the candidate gene *ACTR3* based on the genome-wide association analysis for incident physical fatigue and hippocampal tissue eQTLs (GTEx v7).

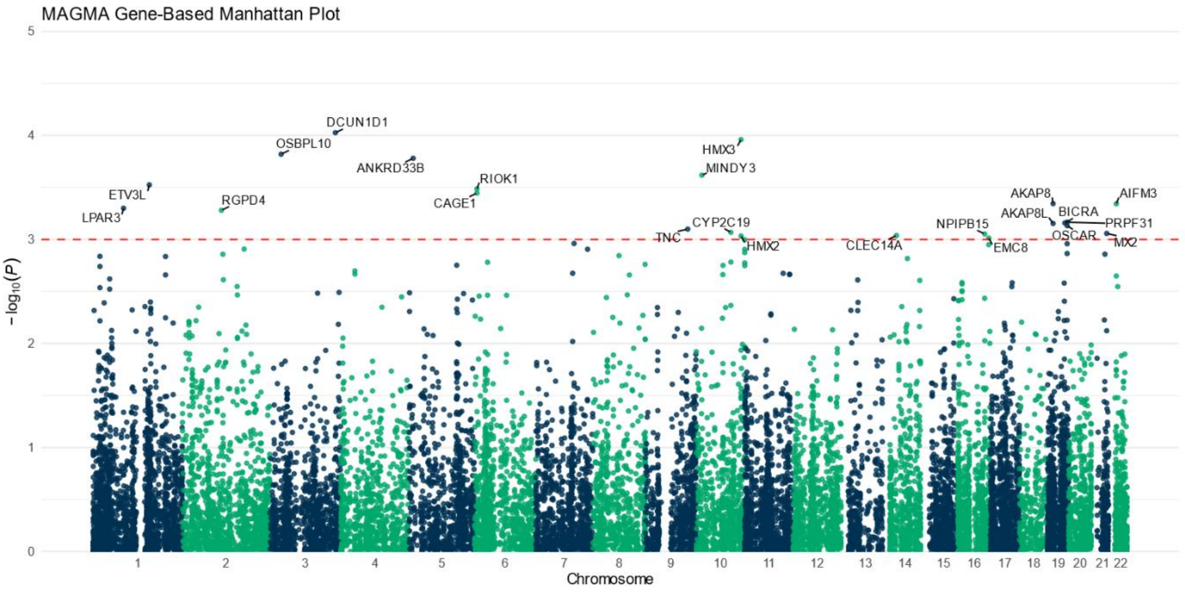

Supplementary figure 11. Manhattan plot for the gene-based association analysis (unadjusted *P*<0.001) using the summary statistics of the genome-wide association analysis on incident physical fatigue up to two years following external beam radiotherapy therapy in prostate cancer patients of the REQUITE cohort without fatigue before the start of radiotherapy.

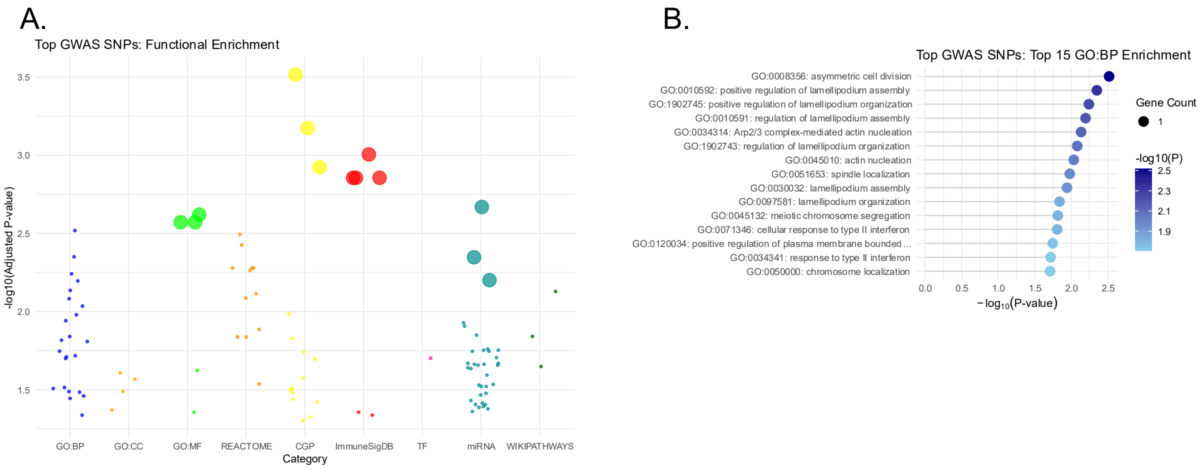

Supplementary figure 12. Functional enrichment analysis of candidate genes mapped from the locus in proximity to the top GWAS-associated SNP rs142212041 on chromosome 2. (A) Over-representation testing across multiple databases. (B) Biological Process Gene Ontology enrichment.

Supplementary table 3. Results of the differential gene expression analysis by SNP-based genotypes for incident long-term physical fatigue in the REQUITE cohort (separate from the GWAS sample).

Gene	Log_2 fold change	Standard error	*P*	P_{adj}
CBWD2	-0.17	0.08	0.04	0.24
ACTR3	-0.09	0.05	0.04	0.24
MIR4782	0.68	0.52	0.19	0.76
FOXD4L1	-0.21	0.44	0.63	0.90
PGM5P4	0.51	1.14	0.66	0.90
WASH2P	0.17	0.24	0.48	0.90
RABL2A	-0.04	0.14	0.78	0.90
ACRP1	-0.90	4.18	0.83	0.90
SLC35F5	-0.03	0.05	0.64	0.90
ACTR3-AS1	-0.06	0.24	0.80	0.90
LINC01191	0.05	0.12	0.68	0.90
AC110769.3	-0.003	0.18	0.99	0.99
