# Supplementary material for: Genetic determinants of fatigue up to 2 years after radiotherapy in prostate cancer patients
Source: Nat Commun. 2026 Apr 22;17:3703. doi: 10.1038/s41467-026-72041-3 (PMC13102951; doi:10.1038/s41467-026-72041-3)
Supplement: Supplementary file 3 — Reporting Summary [file 41467_2026_72041_MOESM3_ESM.pdf]

nature portfolio

Corresponding author(s): Dr. Petra Seibold

Last updated by author(s): Jan 28, 2026

Reporting Summary

Nature Portfolio wishes to improve the reproducibility of the work that we publish. This form provides structure for consistency and transparency in reporting. For further information on Nature Portfolio policies, see our Editorial Policies and the Editorial Policy Checklist.

Statistics

For all statistical analyses, confirm that the following items are present in the figure legend, table legend, main text, or Methods section.

n/a	Confirmed	
☐	☒	The exact sample size (*n*) for each experimental group/condition, given as a discrete number and unit of measurement
☐	☒	A statement on whether measurements were taken from distinct samples or whether the same sample was measured repeatedly
☐	☒	The statistical test(s) used AND whether they are one- or two-sided<br>*Only common tests should be described solely by name; describe more complex techniques in the Methods section.*
☐	☒	A description of all covariates tested
☐	☒	A description of any assumptions or corrections, such as tests of normality and adjustment for multiple comparisons
☐	☒	A full description of the statistical parameters including central tendency (e.g. means) or other basic estimates (e.g. regression coefficient) AND variation (e.g. standard deviation) or associated estimates of uncertainty (e.g. confidence intervals)
☐	☒	For null hypothesis testing, the test statistic (e.g. *F*, *t*, *r*) with confidence intervals, effect sizes, degrees of freedom and *P* value noted<br>*Give P values as exact values whenever suitable.*
☒	☐	For Bayesian analysis, information on the choice of priors and Markov chain Monte Carlo settings
☒	☐	For hierarchical and complex designs, identification of the appropriate level for tests and full reporting of outcomes
☒	☐	Estimates of effect sizes (e.g. Cohen's *d*, Pearson's *r*), indicating how they were calculated

Our web collection on statistics for biologists contains articles on many of the points above.

Software and code

Policy information about availability of computer code

Data collection	OpenClinica, LabKey
Data analysis	PLINK 2.0 was used for the preparation of genetic data and regression modelling.<br>R 4.3.1 and the package qqman were used for creating Manhattan and QQ plots.<br>Bootstrap odds ratios and standard errors were calculated using a parametric bootstrap approach and 3,000 replicates; 95% confidence intervals were computed using conditional likelihood methods as implemented in the R package winnerscurse.<br>Fine-mapping was conducted using the Sum of Single Effects model and the package susieR.<br>Local genetic correlation rg was calculated using the package LAVA.<br>Colocalisation was explored using multi-tissue eQTL data derived from brain (hippocampus) and skeletal muscle tissue and whole blood (Genotype-Tissue Expression (GTEx) project v7); the analysis was conducted using the package eQTpLot.<br>Functional annotation was performed for regions harbouring genome-wide association signals, candidate causal variants, and variants with $r^2 \geq 0.8$ using HaploReg v4.2 and Roadmap data.<br>Gene-based associations were assessed using MAGMA (v1.10) with GWAS summary statistics.<br>Over-representation testing was conducted using clusterProfiler, ReactomePA, and related R packages across multiple databases, including Gene Ontology (biological process, cellular component, molecular function), KEGG, Reactome, and curated MSigDB collections (BIOCARTA, CGP, ImmuneSigDB, transcription factor and microRNA targets, WikiPathways).<br>Heritability analysis was conducted using the package GCTA.<br>The differential gene expression analysis was performed using DESeq2.<br>Linkage disequilibrium (LD), recombination rate, and nearby genes of regions with genome-wide statistically significant SNPs were visualised using LocusZoom.

For manuscripts utilizing custom algorithms or software that are central to the research but not yet described in published literature, software must be made available to editors and reviewers. We strongly encourage code deposition in a community repository (e.g. GitHub). See the Nature Portfolio guidelines for submitting code & software for further information.

Data

Policy information about availability of data

All manuscripts must include a data availability statement. This statement should provide the following information, where applicable:

- Accession codes, unique identifiers, or web links for publicly available datasets
- A description of any restrictions on data availability
- For clinical datasets or third party data, please ensure that the statement adheres to our policy

External research projects may request access to the data analysed in this study, specifically for the purpose of cancer research. Access is granted following an application and approval process managed by the REQUITE Publication Committee, which evaluates requests based on criteria such as alignment with patient consent and applicable data protection laws, and other relevant requirements. All remaining data is available in the article, supplementary material and source data files. Commercial use of the dataset is strictly prohibited. Fees may apply. Contact requite@leicester.ac.uk for more information. Source data are provided with this paper.

Research involving human participants, their data, or biological material

Policy information about studies with human participants or human data. See also policy information about sex, gender (identity/presentation), and sexual orientation and race, ethnicity and racism.

Reporting on sex and gender	Only men (prostate cancer patients) were included.
Reporting on race, ethnicity, or other socially relevant groupings	Participants with <80% European ancestry were excluded for the genome-wide association study.
Population characteristics	European and US population.
Recruitment	Data from the prospective, international patient cohort REQUITE were analysed. 1,760 men with non-metastatic PCa were enrolled from 17 radiation oncology departments in seven European countries and the USA between 2014 and 2016 before the start of radiotherapy.
Ethics oversight	Local ethics committees approved the study.

Note that full information on the approval of the study protocol must also be provided in the manuscript.

Field-specific reporting

Please select the one below that is the best fit for your research. If you are not sure, read the appropriate sections before making your selection.

☒ Life sciences ☐ Behavioural & social sciences ☐ Ecological, evolutionary & environmental sciences

For a reference copy of the document with all sections, see nature.com/documents/nr-reporting-summary-flat.pdf

Life sciences study design

All studies must disclose on these points even when the disclosure is negative.

Sample size	Based on the REQUITE prostate cancer patient cohort of 1,760 men who received radiotherapy for prostate cancer, patients with available genome-wide genotypes who received external beam radiotherapy without brachytherapy and completed a fatigue assessments using the EORTC QLQ-C30 and/or Multidimensional Fatigue Inventory (MFI) questionnaire one and/or two years after radiotherapy were analysed. Data on the fatigue scale from the EORTC QLQ-C30 questionnaire were available for 1,381 patients with available genetic data, with 877 patients reporting at least one of the five fatigue dimensions in the MFI questionnaire at one and/or two years after the end of radiotherapy
Data exclusions	Participants with available genome-wide genotypes were included in the analysis if they received external beam radiotherapy only (therefore, excluding those who received brachytherapy or a brachytherapy boost) and provided at least one EORTC QLQ-C30 fatigue scale and/or MFI dimension assessment one and/or two years after radiotherapy.
Replication	To our knowledge, there is currently no prostate cancer cohort worldwide with MFI and genome-wide genotyping data and prospective long-term follow-up available.
Randomization	n/a
Blinding	Blinding was not relevant for the study investigating genetic variants as determinants of long-term fatigue.

Reporting for specific materials, systems and methods

We require information from authors about some types of materials, experimental systems and methods used in many studies. Here, indicate whether each material, system or method listed is relevant to your study. If you are not sure if a list item applies to your research, read the appropriate section before selecting a response.

Materials & experimental systems

n/a	Involved in the study
☒	☐ Antibodies
☒	☐ Eukaryotic cell lines
☒	☐ Palaeontology and archaeology
☒	☐ Animals and other organisms
☐	☒ Clinical data
☒	☐ Dual use research of concern
☒	☐ Plants

Methods

n/a	Involved in the study
☒	☐ ChIP-seq
☒	☐ Flow cytometry
☒	☐ MRI-based neuroimaging

Clinical data

Policy information about clinical studies

All manuscripts should comply with the ICMJE guidelines for publication of clinical research and a completed CONSORT checklist must be included with all submissions.

Clinical trial registration	https://www.controlled-trials.com ISRCTN98496463
Study protocol	On request.
Data collection	1,760 men with non-metastatic PCa were enrolled from 17 radiation oncology departments in seven European countries and the USA between 2014 and 2016 before the start of radiotherapy.
Outcomes	EORTC QLQ-C30, MFI

Plants

Seed stocks	*Report on the source of all seed stocks or other plant material used. If applicable, state the seed stock centre and catalogue number. If plant specimens were collected from the field, describe the collection location, date and sampling procedures.*
Novel plant genotypes	*Describe the methods by which all novel plant genotypes were produced. This includes those generated by transgenic approaches, gene editing, chemical/radiation-based mutagenesis and hybridization. For transgenic lines, describe the transformation method, the number of independent lines analyzed and the generation upon which experiments were performed. For gene-edited lines, describe the editor used, the endogenous sequence targeted for editing, the targeting guide RNA sequence (if applicable) and how the editor was applied.*
Authentication	*Describe any authentication procedures for each seed stock used or novel genotype generated. Describe any experiments used to assess the effect of a mutation and, where applicable, how potential secondary effects (e.g. second site T-DNA insertions, mosiacism, off-target gene editing) were examined.*
