# Supplementary material for: Genetic determinants of fatigue up to 2 years after radiotherapy in prostate cancer patients
Source: Nat Commun. 2026 Apr 22;17:3703. doi: 10.1038/s41467-026-72041-3 (PMC13102951; doi:10.1038/s41467-026-72041-3)
Supplement: Supplementary file 4 — Description of Additional Supplementary Files [file 41467_2026_72041_MOESM4_ESM.pdf]

Description of Additional Supplementary Files

Supplementary Data 1

Description: Selected characteristics of prostate cancer patients with available genetic data from the REQUITE cohort that assessed the EORTC QLQ-C30 Fatigue Scale and/or at least one Multidimensional Fatigue Inventory dimension up to two years following external beam radiotherapy by clinically important fatigue levels before the start of radiotherapy.

Supplementary Data 2

Description: SNPs (P<5e-06) associated with clinically important fatigue up to two years after the end of RT in prostate cancer patients of the REQUITE cohort. Multivariable regression models were adjusted for age and BMI at enrolment, baseline fatigue level prior to radiotherapy, depression, hormone therapy, prostatectomy, tumour size, nodal involvement, pelvic radiotherapy, and the top 5 principal components.

Supplementary Data 3

Description: SNPs (P<5e-06) associated with clinically important incident fatigue up to two years after the end of RT in prostate cancer patients of the REQUITE cohort without clinically important fatigue prior to radiotherapy. Multivariable regression models were adjusted for age and BMI at enrolment, depression, hormone therapy, prostatectomy, tumour size, nodal involvement, pelvic radiotherapy, and the top 5 principal components.

Supplementary Data 4

Description: Significant results from the functional enrichment analysis of candidate genes mapped from the locus in proximity to the top GWAS-associated SNP rs142212041 on chromosome 2.
